# Efficacy and Safety of Albendazole Versus Albendazole and Ivermectin in Reduction of Soil-Transmitted Helminth Infections in School-Age Children (DVERMIN): A Double-Blind, Randomized, Controlled Trial

**DOI:** 10.7759/cureus.79152

**Published:** 2025-02-17

**Authors:** Shally Awasthi, Mausumi Nandy, Kalpana Datta, Gitika, Ravinder Yadav, Rajat Singal, Monjori Mitra

**Affiliations:** 1 Pediatrics, King George's Medical University, Lucknow, IND; 2 Pediatrics, Medical College Kolkata, Kolkata, IND; 3 Medical Affairs, Mankind Pharma Ltd., New Delhi, IND; 4 Pediatrics, Institute of Child Health (ICH) Hospital, Kolkata, IND

**Keywords:** albendazole, ivermectin, randomized controlled trial, school children, soil-transmitted helminth (sth)

## Abstract

Background: Soil-transmitted helminth (STH) infections are endemic in India despite considerable improvements in hygiene conditions and regular deworming. This multicentric, double-blind, randomized controlled trial (Efficacy and Safety of Albendazole Versus AlbenDazole and IVERmectin in Reduction of Soil-Transmitted HelMINth Infections in School-Age Children (DVERMIN)) was conducted to evaluate the efficacy and safety of albendazole versus albendazole + ivermectin in the treatment of STH infections and to evaluate the prevalence of stool-positivity for STH in school-going children from 12 schools of Lucknow and Kolkata.

Methods: This was a randomized controlled trial conducted from March 2022 to February 2023. The included children aged 6-15 years were administered single-dose oral suspension of albendazole (400 mg) or albendazole (400 mg) + ivermectin (3/6/12 mg based on body weight). The primary endpoint was the microbiological cure rate (proportion of infected children having egg-free stool samples post single-dose treatment) on Day 7. The secondary endpoints were egg reduction rate and clinical cure rate (proportion of children with symptom resolution) on Day 7, and the proportion of children in the regions of study conduct with stool positivity for STH at baseline. Safety was assessed based on adverse events (AEs).

Results: Among the 1233 eligible children, stool samples from 1067 were evaluable at baseline. A total of 416 (38.99%) of these 1067 samples were egg-positive. *Ascaris lumbricoides* was the most prevalent STH (99.52% samples). Of the 416 samples, 304 were evaluable for post-dose analysis (albendazole, 146; albendazole + ivermectin, 158). In these samples, the microbiological cure rate achieved with albendazole (61.64%) was comparable to that achieved with albendazole + ivermectin (62.03%; p=0.95). The egg reduction rate obtained with albendazole was 90.61% compared to 93.22% achieved with albendazole + ivermectin. The rate of complete clinical cure on Day 7 was 66.95% with albendazole and 71.31% with albendazole + ivermectin (p=0.47). A moderate AE unrelated to antihelminthic medication was observed with albendazole; no AE was observed with albendazole + ivermectin.

Conclusions: Overall, both treatments were found to be effective and safe in school-age children. The rates of microbiological cure, egg reduction, and clinical cure were not significantly different between the groups; however, a clinical advantage was demonstrated in the albendazole + ivermectin group with respect to egg reduction and clinical cure.

## Introduction

Soil-transmitted helminth (STH) infections are one of the most common infections with an estimated 1.5 billion infected people globally, accounting for approximately 24% of the world population. Tropical and subtropical regions of Asia are among the places with the highest prevalence due to inadequate levels of sanitation, clean water, and hygiene conditions [1]. Although STH infections are not uncommon in adults, these are more prevalent in preschool and school-age children [2]. According to the World Health Organization (WHO) in 2021, 37.19% of all children requiring preventive chemotherapy for STH globally belonged to India, the only country with >100 million infected children aged 1-14 years [3].

STH refers to intestinal worms that infect humans and are transmitted through contaminated soil. These are typically from the families of nematodes and can be classified as roundworms (e.g., *Ascaris lumbricoides*), whipworms (e.g., *Trichuris trichiura*), hookworms (e.g., *Ancylostoma duodenale* and *Necator americanus*), and threadworms (e.g., *Enterobius vermicularis*). These cause immense disability; however, they can be controlled or eliminated by adopting appropriate measures [4]. Morbidity correlates with the burden of infestation: a few worms usually do not cause any severe symptoms whereas heavy infections can cause diarrhea, abdominal pain, malnutrition, general malaise, and weakness. Infection in children can cause nutritional and/or physical impairment [1], and deficits in development [5]. Therefore, several nations perform regular deworming in children, India being no exception. Since 2015, February 10 has been observed as the National Deworming Day in India to spread awareness regarding the health threats of worm infection and to promote deworming among children aged 1-19 years.

Despite adopting these measures coupled with improving cleanliness and hygiene conditions, reports on STH prevalence in India from 2014 to 2023 have pointed to an endemic nature of these infections in the country [6-12]. This scenario necessitates treatment of STH infections in addition to mass deworming. Albendazole, a medication approved by the United States Food and Drug Administration (FDA) for the treatment of several parasitic worm infections, inhibits microtubule polymerization in a helminth and impairs metabolism [13]. Ivermectin, another antiparasitic medication, targets glutamate-gated chloride channels and other invertebrate neurotransmitter receptors, as well as impairs reproduction-associated gene expression in female worms [14]. Earlier studies from India have proven the efficacy of albendazole in reducing the risk of stunting [15] and improving weight [16,17] in children. The combination of albendazole and ivermectin has been previously reported to result in a favorable cure rate and egg reduction rate in STH infections [18]; the addition of ivermectin has been shown to improve the therapeutic outcome of albendazole [19]. Community-based deworming with this combination was shown to be effective in the reduction of STH prevalence and morbidity [20].

Despite the known efficacy of albendazole and ivermectin, there is a dearth of data from India using albendazole and ivermectin for the treatment of STH infections. Therefore, this study (Efficacy and Safety of Albendazole Versus AlbenDazole and IVERmectin in Reduction of Soil-Transmitted HelMINth Infections in School-Age Children (DVERMIN)) aimed to compare the efficacy and safety of combined albendazole and ivermectin treatment with that of albendazole monotherapy. Additionally, the study evaluated the prevalence of stool positivity for STH in children from 12 schools in the Indian cities of Lucknow and Kolkata. The current study takes advantage of the availability of the test medication, a fixed-dose combination (FDC) of albendazole and ivermectin (Mankind Pharma Ltd., New Delhi, India).

## Materials and methods

This was a double-blind, parallel, randomized controlled trial (RCT) conducted from March 2022 to February 2023 at two sites in India, King George Medical University (Lucknow, Uttar Pradesh) and Calcutta Medical College (Kolkata, West Bengal). Participating children were recruited from a total of 12 schools (seven in Lucknow and five in Kolkata). The study was approved by the Institutional Ethics Committee, King George's Medical University, Lucknow (reference number: ECR/262/Inst/UP/2013/RR-16 dated November 15, 2021) and the Institutional Ethics Committee for Human Research, Medical College Kolkata, Kolkata (reference number: MC/KOL/IEC/SPON/1201/10/21 dated October 8, 2021).

The study was conducted in accordance with the principles of Declaration of Helsinki and Good Clinical Practice (GCP) guidelines for clinical trials on pharmaceutical products in India, as mentioned in New Drugs and Clinical Trials (NDCT) Rules 2019, issued by the Central Drugs Standard Control Organization (CDSCO). The trial was registered with the Clinical Trials Registry-India (CTRI) on January 27, 2022 (reference number: CTRI/2022/01/039738). Participants were enrolled after obtaining written informed parental consent. Verbal assent was obtained from children aged 7-12 years and written assent from those aged 12-15 years.

Eligibility criteria

Children aged 6-15 years, studying in classes 1-10 of the 12 selected schools were eligible to participate if parents provided written informed consent for participation. Children with any major severe systemic illness, chronic severe anemia, history of epileptic disorders, visual disturbances, or chronic headache were excluded from the study. Other disqualifiers were current use of anti-epileptics or drugs known to have pharmacological interaction with albendazole or ivermectin, presence of any acute illness, fever, and vomiting at enrollment, use of anti-helminthic drugs by either the child or any household contact within three months before enrollment, and known allergy to study medications.

Study endpoints

The primary endpoint was the microbiological cure rate, defined as the proportion of children with egg-free post-dose stool samples. The secondary endpoints were as follows: (i) egg reduction rate, evaluated as ((GM EPG at baseline among infected children − GM EPG on Day7)/GM EPG at baseline among infected children) ×100’, where GM refers to geometric mean and EPG refers to eggs per gram, (ii) clinical cure rate defined as the proportion of children with symptom resolution on Day 7, and (iii) prevalence of stool-positivity for STH. Safety assessment was based on adverse events (AEs). The proportion of children with clinical signs of re-emergence of infection on Day 28 was an exploratory endpoint.

Study procedure

Trained personnel from study sites explained the study objectives to the responsible authorities of selected schools and obtained their permission to conduct the trial. The site team collected the contact details of participants’ parents at baseline. Demographic characteristics and symptoms of worm infestation (perianal itching, loss of appetite, disturbed sleep, abdominal pain, diarrhea, or presence of worms in stool), if any, were recorded at baseline. Chronic severe anemia was determined at baseline as per the investigator's decision based on previous reports or clinical assessment of pallor during physical examination.

Each enrolled child was given a clean, dry, leak-proof stool-collection container with a unique identifier. The method of stool collection was explained to the child and parent(s). On the next day (Day 0), stool samples were collected from enrolled children, transported in a cooler box to a central laboratory, and examined independently by two technicians within 24 hours of collection. WHO-recommended Kato-Katz (Sterlitech Corporation, Auburn, Washington, United States) method [21] was used (one slide per sample) following the manufacturer’s instructions. For quality control, all egg-positive fecal smears and 10% of all smears devoid of eggs (egg-free) were re-examined by a third technician.

The number of eggs per slide×24 was recorded as EPG. Enrolled children were randomized in a 1:1 allocation ratio (based on a computer-generated randomization list) to receive a single dose of either albendazole (400 mg) or FDC of albendazole (400 mg) + ivermectin (Mankind Pharma Ltd.) after breakfast. The test FDC was administered based on the dosage of ivermectin recommended as per body weight (15-29 kg, 3 mg; 30-60 kg, 6 mg; >60 kg, 12 mg). The medications were administered orally by trained designated personnel.

To ensure blinding for both the investigator and the children, drug dispensing and administration were done by two different people and both medications had similar flavor, appearance, labels, and packaging. A post-dose stool sample was collected on Day 7 (i.e., seven days after single-dose treatment) from children diagnosed as infected at baseline. Symptom resolution was assessed on Day 7. A single dose of FDC albendazole (400 mg) + ivermectin (12 mg) was administered to the household contacts of infected children on Day 7. On Day 28, children were contacted over the phone or at school to inquire about the possible reappearance of symptoms.

Sample size

Assuming a mean inter-group difference of 20% in microbiological cure rate [22], the sample size was calculated as 101 for each treatment group, considering equal allocation rates, 80% power, 5% level of significance (two-tailed), and 10% dropout rate. It was originally decided to have an equal distribution of children stratified by age groups 6-9, 10-13, and 14-15 years; therefore, 303 children in each group and a total of 606 evaluable children in both groups were planned. The study protocol was prepared before the SARS-CoV-2 pandemic; however, following the outbreak, we faced delays in obtaining study approval from Ethics Committees. Recruitment was initiated when schools reopened after the pandemic. Due to hindrances faced in study conduct, the sample size needed to be revised. Based on the observation that 41.87% of stool samples were egg-positive (containing eggs) among the initial few samples tested at King George Medical University (Lucknow), the number of egg-positive samples required was revised to 416 (considering 95% confidence interval (CI), 5% margin of error, and 10% dropout rate). Screening and enrollment were continued till 416 positive samples were obtained. The planned age-stratified enrollment was not practically possible.

Statistical methods

All statistical methods were based on the International Council for Harmonization E9 document ‘Statistical Principles for Clinical Trials’ and all statistical analyses were done using IBM SPSS Statistics for Windows, Version 28.0.1.1 (2022; IBM Corp., Armonk, New York, United States). All hypothesis testing was conducted at a significance level of 5% (2-sided), with p<0.05 considered statistically significant. Demographic characteristics were assessed using a t-test, while categorical data were analyzed with a chi-square test. The Mann-Whitney U test was employed to compare egg reduction between treatment groups, and the Wilcoxon signed rank test was utilized for pairwise comparisons of egg reduction from baseline to Day 7 within each treatment group.

## Results

**Figure 1:**
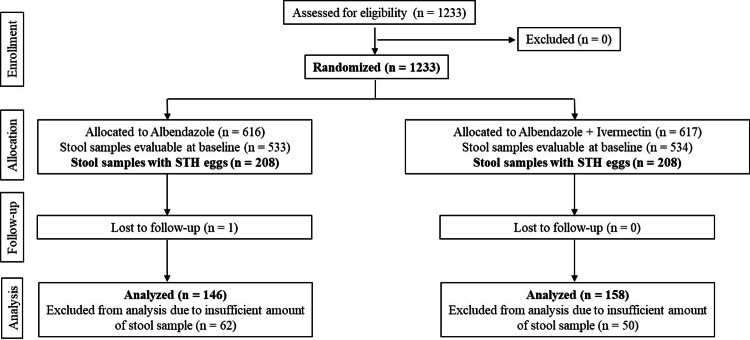
Study flowchart A total of 1233 children were screened and enrolled in this study. Of these, stool samples from 1067 children (533 in albendazole and 534 in albendazole + ivermectin) were evaluable at baseline; 416 of the evaluable samples (208 in each treatment group) were egg-positive. Among these, 304 samples were evaluable (146 in albendazole and 158 in albendazole + ivermectin) for post-dose analysis. NOTE: The lost to follow-up numbers mentioned in Figure 1 are based on Day 28. If the child was not available at the school for this visit, information about the possible reappearance of symptoms was collected over a telephone call.

A total of 1233 eligible children were enrolled in the study. Out of these, stool samples from 1067 children (86.54%) were evaluable at baseline. The remaining children either did not submit stool samples or provided insufficient quantities that did not meet the necessary quality standards. Among the evaluable samples, 416 tested positive for eggs (Figure 1).

Thus, the overall STH prevalence based on stool positivity was 416/1067 (38.99%) with 95%CI (36.06%, 41.99%). The prevalence at Lucknow was 304/588 (51.70%) and that at Kolkata was 112/479 (23.38%). Out of the total 416 samples, 414 (99.52%) contained *A. lumbricoides*, while only two samples contained *Hymenolepis nana*. Of 416 examined samples, 371 (89.18%), demonstrated an egg count ≤10 EPG. Notably, 40 samples (9.61%) had up to 70 EPG, while four samples (0.96%) exhibited EPG values in the range of 190-420. EPG data for one egg-positive sample was unavailable.

Of the 416 infected children, 208 each were treated with albendazole or albendazole + ivermectin. A total of 240/416 (57.69%) children were symptomatic, that is, had at least one symptom of worm infestation at baseline. Among 240 children, 118 were treated with albendazole and 122 with albendazole + ivermectin. ‘Abdominal pain’ was the most common symptom, affecting 162/416 (38.94%) children, followed by ‘loss of appetite’ in 124/416 (29.81%) children. At baseline, the distribution of symptoms and demographic characteristics was similar in both groups (Table 1).

**Table 1:** Baseline characteristics of children with egg-positive stool samples in both treatment groups Each enrolled child presented with at least one symptom of intestinal worm infestation at baseline. NA: not applicable

	Albendazole (n = 208)	Albendazole + Ivermectin (n = 208)	t-value	χ^2^-value	p-value
Age (years)					
Mean ± SD	9.14 ± 2.40	9.25 ± 2.40	-0.47	NA	0.64
Median (min, max)	9 (6, 15)	9 (6, 15)			
Height (cm)					
Mean ± SD	127.86 ± 17.01	127.38 ± 15.55	0.30	NA	0.76
Median (min, max)	128.15 (44, 169)	128 (82, 161)			
Weight (kg)					
Mean ± SD	28.09 ± 10.03	27.27 ± 9.41	0.86	NA	0.39
Median (min, max)	25.85 (14, 63)	25 (15, 71)			
Temperature (°F)					
Mean ± SD	97.85 ± 0.76	97.92 ± 0.68	-1.01	NA	0.31
Median (min, max)	98.10 (93, 99)	98.10 (95, 99)			
Proportion of children in different age groups, n (%)					
6-9 years	122 (58.65%)	114 (54.81%)	NA	0.63	0.43
10-13 years	76 (36.54%)	86 (41.35%)	NA	1.01	0.31
14-15 years	10 (4.81%)	8 (3.85%)	NA	0.23	0.63
Sex, n (%)					
Female	105 (50.48%)	108 (51.92%)	NA	0.09	0.77
Male	103 (49.52%)	100 (48.08%)			
Symptoms of intestinal worm infestation, n (%)					
Abdominal pain	83 (39.90%)	79 (37.98%)	NA	0.16	0.69
Loss of appetite	58 (27.88%)	66 (31.73%)	NA	0.74	0.39
Perianal itching	33 (15.87%)	27 (12.98%)	NA	0.70	0.40
Presence of worms in stool	19 (9.13%)	12 (5.77%)	NA	1.71	0.19
Diarrhea	13 (6.25%)	16 (7.69%)	NA	0.33	0.56
Disturbed sleep	15 (7.21%)	9 (4.33%)	NA	1.59	0.21

In the post-dose analysis, 304/416 (73.08%) samples were evaluable. Among 304 samples, 146 were assigned to the albendazole group, while 158 were included in the albendazole + ivermectin group. The microbiological cure rates were comparable between the two treatment groups, with 90/146 (61.64%) achieving cure in the albendazole group and 98/158 (62.03%) in the albendazole + ivermectin group (p=0.95) (Table 2).

**Table 2:** Rates of microbiological cure, clinical cure, and symptom reappearance in both treatment groups #The denominator denotes the number of evaluable stool samples on Day 7. §The denominator denotes the number of children with each symptom at baseline. ¥ The denominator denotes the number of children with both symptoms at baseline. €The denominator denotes the number of children with at least one symptom at baseline.

Efficacy variable	Albendazole, n (%)	Albendazole + Ivermectin, n (%)	χ^2^-value	p-value
Microbiological cure rate on Day7^#^	90/146 (61.64%)	98/158 (62.03%)	0.00	0.95
Clinical cure rate for each symptom on Day7^§^				
Abdominal pain	75/83 (90.36%)	74/79 (93.67%)	0.60	0.44
Loss of appetite	28/58 (48.28%)	40/66 (60.61%)	1.90	0.17
Perianal itching	29/33 (87.88%)	25/27 (92.59%)	0.36	0.54
Presence of worms in stool	19/19 (100.00%)	12/12 (100.00%)	0.00	0.99
Diarrhea	12/13 (92.31%)	15/16 (93.75%)	0.02	0.88
Disturbed sleep	15/15 (100.00%)	8/9 (88.89%)	1.74	0.19
Clinical cure rate for predominant symptoms (‘abdominal pain’ and ‘loss of appetite’) on Day7^¥^	15/37 (40.54%)	20/38 (52.63%)	1.10	0.29
Rate of complete clinical cure (resolution of all symptoms present at baseline) on Day7^€^	79/118 (66.95%)	87/122 (71.31%)	0.54	0.47
Rate of symptom reappearance on Day28	7/208 (3.37%)	10/208 (4.81%)	0.55	0.46

The EPG decreased significantly from baseline to Day 7 in both groups (p<0.001). In subjects with reduced EPG after treatment, the egg reduction rate was 90.61% for the albendazole group and 93.22% for the albendazole + ivermectin group (Table 3).

**Table 3:** Egg reduction rate in the two treatment groups ***: p<0.001 EPG: eggs per gram; Q1: lower quartile; Q3: upper quartile

	Albendazole (n = 122)	Albendazole + Ivermectin (n = 117)	Mann-Whitney U value	p-value between groups
EPG at Baseline				
Arithmetic mean ± SD	6.02 ± 9.78	5.21 ± 9.72	6621.50	0.33
Median (Q1, Q3)	3 (2, 5)	3 (2, 4)		
Geometric mean	3.62	2.95		
EPG at Day 7				
Arithmetic mean ± SD	0.73 ± 2.29	0.37 ± 0.91	6566.00	0.15
Median (Q1, Q3)	0 (0, 1)	0 (0, 0)		
Geometric mean	0.34	0.20		
Egg reduction from Baseline to Day 7				
Difference in Arithmetic mean	5.30	4.85	6988.50	0.78
Median of difference (Q1, Q3)	2 (1, 4)	2 (1, 3)		
Egg reduction rate (%)	90.61%	93.22%		
Z value	-9.64	-9.45		
p-value within each treatment group	<0.001***	<0.001***		

Among symptomatic children, 166/240 (69.17%) achieved complete symptom resolution, the proportion in the albendazole group being 79/118 (66.95%) and that in albendazole + ivermectin group being 87/122 (71.31%) (p=0.47). The clinical cure rate for each symptom was not significantly different between the treatment groups: the presence of worms in stool was resolved in all children from both groups; disturbed sleep was cured in all children in the albendazole group and in 8/9 (88.89%) children in the albendazole + ivermectin group; perianal itching, loss of appetite, abdominal pain, and diarrhea were cured in up to 12% more children in the albendazole + ivermectin group compared to albendazole. The proportion of children with resolution of both the predominant symptoms (abdominal pain and loss of appetite) was 15/37 (40.54%) in the albendazole group and 20/38 (52.63%) in the albendazole + ivermectin group (p=0.29). The reappearance of symptoms (like loss of appetite, abdominal pain, disturbed sleep) was observed in 3.37% of children in the albendazole group and 4.81% of children in the albendazole + ivermectin group; however, the difference between the groups was not statistically significant (p=0.46) (Table 2).

A moderate AE consisting of vomiting and fever, not related to anti-helminthic medication, was reported for one child in the albendazole group; the AE resolved completely with prescribed medications. No AE was reported in the albendazole + ivermectin group.

## Discussion

This study showed that following single-dose treatment with albendazole or albendazole + ivermectin on Day 0, about 62% of children in each treatment group were devoid of STH eggs on Day 7. A meta-analysis had earlier shown that co-administration of albendazole and ivermectin offered no/marginal advantage in egg reduction and cure rates over albendazole monotherapy against *Ascaris *[19]. Likewise, the current study reports comparable efficacy between albendazole and albendazole + ivermectin treatments based on microbiological cure rate. The egg reduction rate achieved in the current study was approximately 91% with albendazole and 93% with albendazole + ivermectin. About 71% of children in the albendazole + ivermectin group and 67% of children in the albendazole group showed resolution of clinical symptoms of worm infestation on Day 7. Less than 5% of children in each group had symptom reappearance on Day 28. Taken together, both albendazole and albendazole + ivermectin were found to be effective for the management of STH infections in the majority of children in this study.

Data from this study revealed an overall STH prevalence of about 39% in children; almost half of the enrolled children in schools from Lucknow were found infected as compared to one-fourth in Kolkata. A study in rural and urban regions of south India in 2014 revealed up to 20.4% STH prevalence among school children, with *Ascaris *being more prevalent in urban children [6]. A high prevalence of *Ascaris *(69.6%) was reported among school children in Uttar Pradesh in 2015 [10]. *Ascaris *was also identified as the commonest STH in an epidemiological study conducted between November 2015 and January 2016 in three other Indian states with an overall prevalence in each state >51% [11]. Similar was the result from a 2017 systematic review, wherein a prevalence of >20% and >50% was reported in Uttar Pradesh and West Bengal, respectively [7].

The DeWorm3 trial conducted in a south Indian state between December 2017 and February 2018 reported an unweighted prevalence of 17% with *Ascaris *prevalence being <1% [12]. In 2020, STH prevalence in an urban resettlement colony of Delhi was 54.8%, the majority (85.3%) of which was *Ascaris *[9]. As is evident, *Ascaris *prevalence reports vary across states and years, and this is reflected in a recent meta-analysis wherein its pooled prevalence was reported as 25% with considerable heterogeneity, ranging from 0.8% to 91% across reports. Northeastern and northern India had the maximum prevalence (46% and 35%, respectively) [8]. Taken together, prevalence estimates from the current study corroborate earlier reports from India and provide important evidence on the current prevalence to prove that STH infections are persistent in the country despite socioeconomic development, improvement of hygiene conditions, and commendable efforts towards mass deworming in children. India has been classified as an STH-endemic country where preventive chemotherapy has an effective coverage of <5 years [23]. Since infection/non-treatment in children is associated with developmental deficits and deworming has been shown to have cognitive and educational benefits [5], it is crucial to potentiate quick and substantial improvement in the current status of STH infections. Therefore, treatment modalities are required to complement preventive strategies, nationwide deworming, and improvement of hygiene conditions and habits.

Safety analysis showed that only one AE was reported in albendazole and none in the albendazole + ivermectin group in the present study. Cumulatively, findings from this study support the efficacy and safety of STH treatment with albendazole as well as albendazole + ivermectin with about 62% of infected children in each group showing microbiological cures after treatment. However, 31/146 (21.23%) children in albendazole and 35/158 (22.15%) in the albendazole + ivermectin group had reduced EPG, but were not completely egg-free/cured. Repeated doses of medication might be useful to attain a complete cure. The efficacy of repeated dosage of albendazole against *Ascaris *has recently been proven [24]. Such a treatment regimen might benefit children from regions with high STH infestation. Based on the prevalence of STH infections in India, annual or biannual mass deworming as per WHO guidelines is scheduled in different states of India. Considering the high prevalence in India which is also evident in our study (~39%), our objective was to follow the schedule and check if the groups showed any difference with respect to the study endpoints upon treatment with a single dose of medication. Since this study protocol was aligned with the mass deworming strategy used in India currently, the use of multiple doses of medication was beyond the scope of the study. This limitation can be overcome by designing large-scale studies with a provision of multiple dosing. Such studies are expected to strengthen the findings from this study.

The data from this study on STH prevalence is expected to encourage the adoption of precautionary measures. Results from this study on the efficacy and safety of albendazole + ivermectin unveil a possible treatment option that can be explored further. Given the favorable trend of outcomes observed with albendazole + ivermectin coupled with known literature on the benefits of this combination [18-20], its use can be explored for mass deworming. Large population-based studies are warranted to understand the efficacious benefits and recurrence patterns achieved with this FDC.

We faced significant challenges in recruitment and sample collection mostly owing to the altered mindset of people after the COVID-19 pandemic. It was a practical difficulty to enable the collection of stool samples within school premises owing to an apprehension towards the use of biological samples and a fear of imminent virus spread. Furthermore, the study was halted repeatedly because the functioning of schools was not regular after the pandemic. Despite multiple challenges encountered, the large number of children enrolled from multiple schools and the evaluable quality of 86.54% of samples are definite strengths of this study. However, it would have benefitted the study if the sample size and age stratification planned originally could be attained. The sample size used in the study was perhaps not adequate to detect an inter-group difference of less than 20% in microbiological cure rates. Also, only 10.57% of samples had >10 EPG. Therefore, the efficacy of the test FDC in treating high STH infestation remains to be investigated.

## Conclusions

Results from the current study showed that the microbiological cure rate achieved with an FDC of albendazole and ivermectin was comparable to that achieved using albendazole alone. The difference in egg reduction rate and clinical cure rate between the treatment arms was not statistically significant although a clinical advantage was seen with the combinatorial therapy. Both treatments were found to be safe with one AE reported in the study. Taken together, findings from this study showed that similar to albendazole, its combination with ivermectin was effective and safe for STH treatment in school-age children from regions with high STH prevalence.
